# Mode-Dependent Antiviral Activity of Medicinal Plant Extracts against the Mosquito-Borne Chikungunya Virus

**DOI:** 10.3390/plants10081658

**Published:** 2021-08-11

**Authors:** Sze Mun Chan, Kong Soo Khoo, Shamala Devi Sekaran, Nam Weng Sit

**Affiliations:** 1 Department of Allied Health Sciences, Faculty of Science, Universiti Tunku Abdul Rahman, Bandar Barat, Kampar 31900, Malaysia; michellemun17@hotmail.com; 2 Department of Chemical Science, Faculty of Science, Universiti Tunku Abdul Rahman, Bandar Barat, Kampar 31900, Malaysia; khookongsoo@yahoo.co.uk; 3 Faculty of Medicine & Health Sciences, UCSI University, Bandar Springhill, Port Dickson 71010, Malaysia; shamala@ucsiuniversity.edu.my

**Keywords:** sequential solvent extraction, cytotoxicity, real-time RT-PCR, Vero cell, viral load, chikungunya, alphavirus, antiviral

## Abstract

The lack of specific treatment for chikungunya fever makes the need for anti-chikungunya virus agents more crucial. This study was conducted to evaluate 132 extracts obtained by sequential solvent extraction from 21 medicinal plants for cytopathic effect inhibitory activity using virus-infected Vero cells in two different sample introduction modes. Among the extracts, 42 extracts (31.8%) from 12 plants in the concurrent mode and three extracts (2.3%) from a plant in the non-concurrent mode displayed strong cytopathic effect inhibitory activity (cell viability ≥70%). Viral load quantification analysis unveiled that the extracts of *Clinacanthus nutans* (chloroform, ethyl acetate, and ethanol), *Hydrocotyle sibthorpioides* (ethanol), and *Ocimum americanum* (ethanol and methanol) hindered the release of viral progeny from the infected cells while the extracts of *Ficus deltoidea* (ethanol), *Gynura bicolor* (water), *H. sibthorpioides* (water), and *O. americanum* (chloroform and ethyl acetate) blocked the entry of virus into the cells. The extracts of *Diodella sarmentosa* (ethyl acetate), *Diplazium esculentum* (chloroform, ethyl acetate, and ethanol), and *G. bicolor* (ethanol) possessed virucidal effect and caused 5.41-log to 6.63-log reductions of viral load compared to the virus control. The results indicate that these medicinal plants are potential sources of anti-chikungunya virus agents that have varied modes of action.

## 1. Introduction

Chikungunya virus is an enveloped, single-stranded, positive-sense RNA virus belonging to the genus *Alphavirus* of the *Togaviridae* family. It is an arthropod-borne virus causing chikungunya fever in humans [1]. The virus was first isolated from a febrile patient in the southern Tanzania in 1952–1953 [2]. Subsequent outbreaks of chikungunya infection have largely been confined to the countries in sub-Saharan Africa and Asia [3]. However, over the last two decades, the virus has caused devastating epidemics in India, Southeast Asia and Pacific Islands, and islands in the Indian Ocean, leading to over six million cases of infection [4]. Since 2013, the virus has spread and established its autochthonous transmission in the Western Hemisphere, resulting in over two million suspected cases being reported in almost 50 countries in the Americas. The virus has been documented in 114 countries and territories [5]. Recent analysis indicates that this virus caused an average yearly loss of over 106,000 disability-adjusted life years for the period 2010–2019 [6]. According to the surveillance done by the European Centre for Disease Prevention and Control, at least 170,000 cases of chikungunya fever occurred globally in the year 2020 [7]. Malaysia recorded 2556 cases in the same year and about 86% of the cases happened in the states of Perak and Penang [8].

Chikungunya virus is mainly transmitted via a bite by the infected mosquitoes *Aedes aegypti* and *Aedes albopictus*. The virus can cross the placenta and goes into the fetus in the vertical transmission mode, which results in higher rates of infant morbidity [9]. Upon an acute infection, 80–97% of patients are symptomatic [10] and their clinical manifestations include arthralgia or polyarthralgia, high fever, headache, myalgia, skin rashes, joint swelling, and nausea [11]. Although the mortality rate is relatively low (0.07%), polyarthralgia can persist in the patients for several months or even years after resolution of the acute phase of infection, this being the most common long-term sequel of chikungunya virus infection [6,12]. Presently, patients with chikungunya fever are treated with antipyretic, analgesic, or anti-inflammatory drugs for symptomatic relief [5]. While efforts have been pursued to develop safe and effective vaccines for prophylaxis and antiviral drugs for therapeutics [13,14,15], currently there is no licensed vaccine or drug available against chikungunya virus. This emphasizes the need for more antiviral drugs.

According to the World Health Organization estimates, approximately 80% of the world population use medicinal plants for some aspects of primary health care [16]. Plants are able to produce arrays of phytochemicals with diverse chemical structures such as alkaloids, terpenoids, essential oils, flavonoids, and polyphenols. Many of these phytochemicals, which are derived from the different pathways of secondary metabolism, serve as chemical weapons for the plants against microbial infections, predations by insects and herbivores. They may also be indicators of environmental stress [17]. These phytochemicals are also found to have various biological activities which are beneficial to human health. The antiviral activities of medicinal plants have been well documented against human immunodeficiency [18,19], influenza, herpes simplex [20,21], hepatitis [21,22], and dengue viruses [23,24]. A total of 17 extracts are reported to have anti-chikungunya virus activity from the screenings of 84 medicinal or endemic plants [25,26]. Epigallocatechin gallate derived from *Camellia sinensis* and curcumin from *Curcuma longa* are reported to prevent chikungunya virus from attachment to cells [27,28] while harringtonine from *Cephalotaxus harringtonia* is able to block the replication of the virus in vitro [29].

Twenty-one species of medicinal plants belonging to 19 families were selected for the present study and extracted sequentially using six solvents of increasing polarity. The medicinal or folkloric uses of these medicinal plants are shown in Table 1. The phytochemicals are segregated into different extractants based on their polarity and solubility during sequential solvent extraction [30]. Less polar solvents such as hexane and chloroform could extract alkaloids, coumarins, fatty acids, and terpenoids while more polar solvents such as ethyl acetate, ethanol, methanol, and water could yield saponins, tannins, flavones, polyphenols, terpenoids, anthocyanins, polypeptides, and lectins from plants [31]. The objectives of this study were to evaluate the plant extracts for cytopathic effect inhibitory activity using chikungunya virus-infected African monkey kidney epithelial (Vero) cells in two different sample introduction modes, i.e., concurrent and non-concurrent modes. In the concurrent mode, the plant extracts and the virus inoculum were introduced simultaneously to the cells whereas for the non-concurrent mode, the cells were incubated with the extracts for 24 h before the addition of the virus inoculum. The modes of action of the selected active extracts were assessed based on the quantification of viral load using real-time reverse-transcriptase polymerase chain reaction (RT-PCR). The results of this study highlighted that medicinal plant extracts possess anti-chikungunya virus activity with varied modes of action.

## 2. Results and Discussion

A total of 132 extracts obtained from 21 plant species were subjected to the antiviral activity screening against the chikungunya virus. As six different solvents were used, each extractant yielded 22 extracts. The ability of an extract to protect Vero cells from the cytopathic effect caused by the virus was used as a measurement of antiviral activity for the extract. As an extract is a mixture of many phytochemicals extracted from a particular plant part, it may contain compounds that are toxic to Vero cells. Thus, it is necessary to determine the non-toxic concentrations for use in the cytopathic effect inhibitory assay. As such, a standardized test concentration range of an extract is not feasible.

In order to express and classify the cytopathic effect inhibitory activity of an extract, three scales were established based on percentage of cell viability, these being strong inhibitory activity when the cell viability is ≥70%, intermediate inhibitory activity when the cell viability is 31–69%, and weak inhibitory activity when the cell viability is ≤30%. The inhibitory activity for each extract is shown in Table 2. Forty-two extracts (31.8%) were found to have strong inhibitory activity in the concurrent mode compared to only three extracts (2.3%) in the non-concurrent mode. These extracts were derived from 12 medicinal plants, i.e., *Azadirachta indica*, *Clinacanthus nutans*, *Diodella sarmentosa*, *Diplazium esculentum*, *Ficus deltoidea*, *Gynura bicolor*, *Hydrocotyle sibthorpioides*, *Homalocladium platycladum*, *Ocimum americanum*, *Petroselinum crispum*, *Sechium edule*, and *Strobilanthes crispus*. The results indicate that the cytopathic effect inhibitory activity was dependent on plant species and sample introduction mode. The results also suggest that phytochemicals in the extracts could exert an inhibitory effect against the virus in the concurrent mode but lost their activity in the non-concurrent mode. The exposure of Vero cells to the extracts for 24 h before the addition of virus inoculum could result in the metabolism of active phytochemicals into metabolites devoid of inhibitory activity. An exception was noted for the three extracts with strong inhibitory activity in the non-concurrent mode. They were ethyl acetate, ethanol, and methanol extracts of *F. deltoidea*. The corresponding cell viabilities in the concurrent mode were 66.8% ± 4.2% at 10 µg/mL, 71.9% ± 5.3% at 40 µg/mL, and 1.5% ± 2.9% at 40 µg/mL, respectively, and increased to 76.5% ± 4.1% (*p* = 0.046), 90.3% ± 0.8% (*p* = 0.024), and 79.8% ± 6.7% (*p* < 0.001), respectively, in the non-concurrent mode, suggesting that the metabolites produced (in the non-concurrent mode) may have stronger activity than their parent compounds.

**Figure 1:**
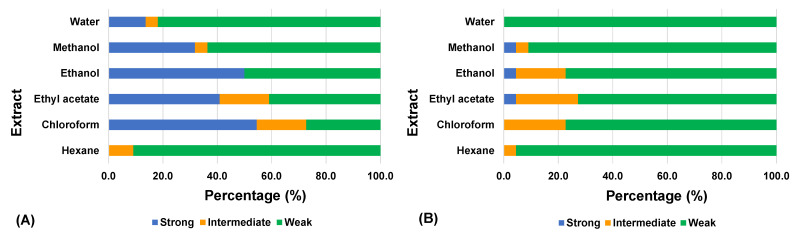
Classification of medicinal plant extracts according to their cytopathic effect inhibitory activity against chikungunya virus in (**A**) concurrent mode and (**B**) non-concurrent mode. The number of extracts for each extractant is 22. The inhibitory activity is measured based on the percentage of viable cells protected by an extract from the cytopathic effect caused by the virus. Strong: cell viability ≥70%; intermediate: cell viability 31–69%; weak: cell viability ≤30%.

The data in Figure 1 indicate that extractants such as chloroform, ethyl acetate, ethanol, and methanol resulted in higher activity compared to hexane and water, both in the concurrent mode and non-concurrent mode. The type of solvent used to extract phytochemicals from plants is an important contributing factor to the results of the bioassay. Phytochemicals of a plant part are solubilized in an extractant based on their polarity [30].

**Figure 2:**
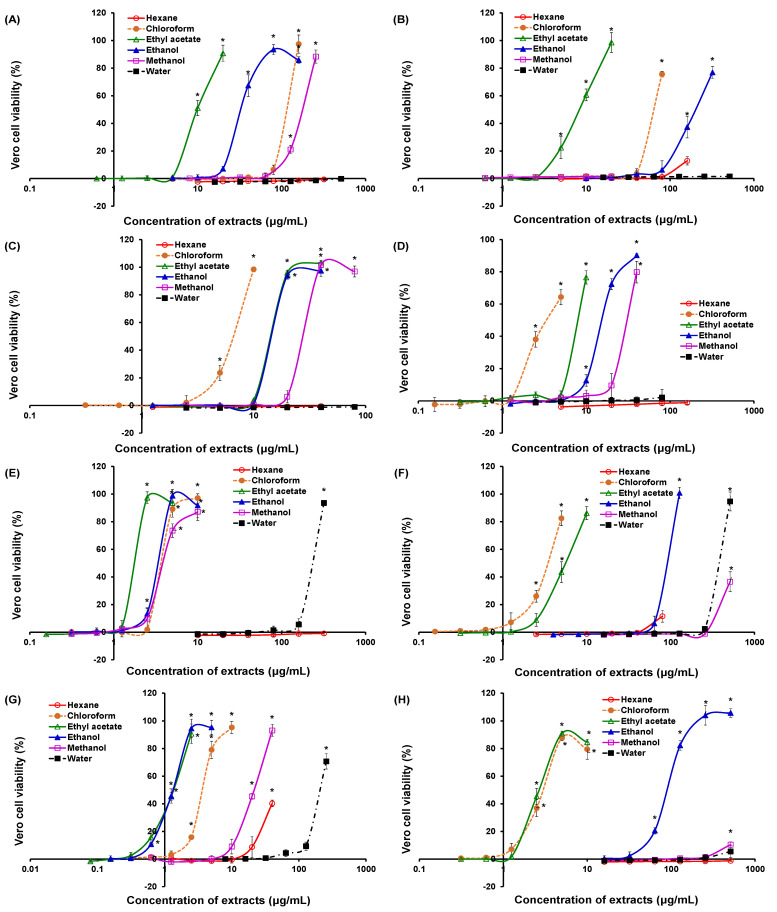
Viability of Vero cells infected by chikungunya virus and treated with different extracts of medicinal plants. (**A**) *Clinacanthus nutans*; (**B**) *Diodella sarmentosa*; (**C**) *Diplazium esculentum*; (**D**) *Ficus deltoidea*; (**E**) *Gynura bicolor*; (**F**) *Hydrocotyle sibthorpioides*; (**G**) *Ocimum americanum*; (**H**) *Sechium edule*. All plants are shown for the concurrent mode except *Ficus deltoidea*, which is in the non-concurrent mode. The cell viability is measured using the neutral red uptake assay. The notated asterisks (*) denote significant differences (*p* < 0.05) among concentrations within an extract by one-way ANOVA.

Among the 45 extracts which exhibited strong inhibitory activity, 20 extracts of seven plants from the concurrent mode and one extract of a plant from the non-concurrent mode were able to protect ≥90% of Vero cells from the cytopathic effect caused by the virus, as shown in Figure 2. These 21 extracts are of great potential for further drug developments. A wide half-maximal effective concentration (EC_50_) range was observed for these extracts, ranging from 1.33 µg/mL for the ethanol extract of *O. americanum* to 394.0 µg/mL for the water extract of *H. sibthorpioides* (Table 3). Consequently, the selectivity indices for these extracts ranged from 2.62 to 170.2. The indices for the ethanol extract of *S. edule* and the water extract of *H. sibthorpioides* could not be calculated as no significant cytotoxicity (*p* > 0.05) was recorded.

In order to yield some indications of the antiviral mechanisms of the 21 extracts, quantification of the viral copy number in the experiments was performed using a real-time RT-PCR. The results are shown in Table 3. All extracts of *C. nutans* and *S. edule*, ethanol extract of *H. sibthorpioides*, and ethanol and methanol extracts of *O. americanum* produced a viral copy number similar to the virus control (*p* > 0.05), suggesting the virus was successfully replicated in the Vero cells but the release of the viral progeny was inhibited by these extracts, and this prevented the occurrence of cytopathic effect in the cells. In contrast, the viral copy numbers for the infected cells treated with the extracts of *F. deltoidea* (ethanol), *G. bicolor* (water), *H. sibthorpioides* (water), and *O. americanum* (chloroform and ethyl acetate) were not significantly different (*p* > 0.05) from the copy number of the viral inoculum. The results suggested that these extracts may work as a fusion inhibitor and block the entry of the virus into the cells. The virus was not able to replicate in the experiments and the copy number remained similar to that of the viral inoculum throughout the 72-h incubation period. The viral copy numbers for five extracts, i.e., ethyl acetate extract of *D. sarmentosa*, chloroform, ethyl acetate, and ethanol extracts of *D. esculentum*, and ethanol extract of *G. bicolor*, were significantly lower (*p* < 0.05) than that of the virus inoculum. These extracts caused 5.41-log to 6.63-log reductions of viral load compared to the virus control (Table 3) as quantified by real-time RT-PCR, suggesting the active phytochemicals in the extracts possessed a virucidal effect on the chikungunya virus. The viral copy number indicates that phytochemicals may have different modes of action against the virus, as illustrated by the extracts of *H. sibthorpioides* and *O. americanum* whereby these extracts could prevent the release of viral progeny and the entry of the virus into cells. Similarly, the extracts of *G. bicolor* could kill the virus, as well as block the virus from entry into the cells.

Chloroquine, which was used as a positive control, is reported to interfere with the protonation of the endocytic vesicles thereby raising the endosomal pH and preventing the fusion of chikungunya virus to the host cell [53]. The EC_50_ value of chloroquine obtained for the non-concurrent mode (9.05 µg/mL or 17.5 µM) was significantly higher (*p* < 0.001) than the concurrent mode (1.92 µg/mL or 3.72 µM) (Table 3), suggesting that the metabolism of chloroquine in the Vero cells may have reduced its efficacy by producing non-active metabolites or metabolites with reduced efficacy against the chikungunya virus. The EC_50_ value (3.72 µM) obtained in this study was generally lower than the values (5.0–11 µM) reported in the literature, probably resulting from the types of cells and the virus strains used [54,55,56].

To the best of our knowledge, this study constitutes the first report of the antiviral properties of the medicinal plants *D. sarmentosa*, *D. esculentum*, *F. deltoidea*, *G. bicolor*, *H. platycladum*, and *S. edule*. *Azadirachta indica*, popularly known as neem, has been extensively used in the Unani, Ayurveda, and Chinese traditional systems of medicine [35]. Raghavendhar et al. studied the water extract of the bark of *A. indica* against chikungunya virus and reported that the extract did not reduce the plaque formation in Vero cells [57]. In contrast, the current study shows that the chloroform, ethyl acetate, ethanol, and methanol extracts of the leaves of *A. indica* had strong cytopathic effect inhibitory activity against the virus (Table 2). For *C. nutans*, *H. sibthorpioides*, *O. americanum*, *P. crispum*, and *S. crispus*, the results of this study further strengthen the case for these plants as potential sources of antiviral compounds. The antiviral activity of *C. nutans* has well been documented against herpes simplex virus types 1 and 2 [58,59], human papillomavirus [60], and dengue virus [61]. The methanol extract and the asiaticoside isolated from *H. sibthorpioides* possess anti-dengue virus activity [62] and anti-hepatitis B virus activity [63], respectively. The dichloromethane and methanol extracts of *O. americanum* and the methanol extract of *S. crispus* displayed anti-herpes simplex virus activities [64,65]. The methanol extract of *P. crispum* has been reported to have inhibitory activity against the Sindbis virus, which like the chikungunya virus is an alphavirus [66]. Further studies need to be carried out to elucidate the identity of antiviral compounds in the active extracts, the inhibitory or virucidal potential for the isolated pure compounds, and the possible synergistic effects among the isolated compounds in targeting different mechanisms of the life cycle of chikungunya virus.

## 3. Materials and Methods

### 3.1. Plant Samples and Extraction

Twenty-one species of medicinal plants were used in the study. A random selection approach was used in sourcing the plant materials which were depended on the accessibility and availability of the materials during the study period. The part used for each plant and the specimen voucher numbers are depicted in Table 1. The seeds and pods of the fruit of *Parkia speciosa* were used as two different parts in the study. The plant samples were sourced from different states of Peninsular Malaysia, i.e., Penang, Perak, Kelantan, Pahang, Selangor, and Johor from March 2010 to August 2011. The identity of the plant samples was ascertained by an ethnobotanist (Professor Hean Chooi Ong) formerly affiliated with the Faculty of Science, Universiti Malaya, Malaysia. The specimen vouchers were deposited in the Faculty of Science, Universiti Tunku Abdul Rahman, Malaysia. After thorough cleaning under running tap water, the fresh plant materials were blended prior to extraction. The extraction was performed sequentially using the analytical grade of solvents hexane (Qrec, Chonburi, Thailand), chloroform (Qrec, Chonburi, Thailand), ethyl acetate (Merck, Darmstadt, Germany), ethanol (Merck, Darmstadt, Germany), methanol (RCI Labscan, Bangkok, Thailand), and lastly distilled water. The plant samples were macerated in each solvent for three cycles (one day per cycle) at room temperature and agitated at 110 rpm using an orbital shaker (IKA-Werke, Staufen, Germany). The filtrates collected after the maceration were concentrated to dryness at 40 °C by rotary evaporation [67]. The dry extracts were stored at −20 °C pending bioassay.

### 3.2. Cell Culture and Virus Cultivation

African monkey kidney epithelial (Vero) cells (ATCC^®^ CCL-81) were used to cultivate chikungunya virus. The cell line was purchased from the American Type Culture Collection (Manassas, VA, USA). The cells were cultured in Dulbecco’s modified Eagle medium (DMEM) (Sigma-Aldrich, St. Louis, MO, USA) supplemented with 10% fetal bovine serum, 10 kU/mL of penicillin, 10 mg/mL of streptomycin, and 3.7 mg/mL sodium bicarbonate at pH 7.4, and maintained at 37 °C in a humidified 5% CO_2_ incubator. The chikungunya virus was provided by the Faculty of Medicine, Universiti Malaya, Malaysia. The virus belongs to the Asian genotype with an accession number of EU703761. Vero cells were inoculated with the virus, incubated at 37 °C and 5% CO_2_, and observed daily for the development of cytopathic effect. The infected cell culture was spun down at 1000 rpm for 10 min at 4 °C. The resulted supernatant was aliquoted and stored in a liquid nitrogen tank. The viral titer of each aliquot was determined based on the median tissue culture infectious dose [68].

### 3.3. Cytotoxic Assay

Each plant extract was evaluated for cytotoxicity using the method of Chan et al. [69] in order to determine the non-toxic concentrations to be used for cytopathic effect inhibitory assay. The plant extract stock solution was prepared in a dimethyl sulfoxide-ethanol mixture (3:2, *v*/*v*) at 256 mg/mL and two-fold serially diluted in the maintenance medium (DMEM with 1% fetal bovine serum) to produce eight concentrations for evaluation. For this purpose, 40,000 Vero cells were seeded in each well of a 96-well microplate and incubated at 37 °C and 5% CO_2_ for 24 h. A volume of 100 µL of the extract was then added and further incubated for 72 h under the same conditions. The final concentration range of each extract was 5–640 µg/mL. Vero cells without any extract treatment were used as a cell control. One hundred µL of 40 µg/mL neutral red solution (Sigma-Aldrich, St. Louis, MO, USA) was added into each well to examine the cell viability. After two hours of incubation, the medium in each well was replaced with 150 µL of neutral red destain solution (ethanol:glacial acetic acid:water, 50:1:49, *v*/*v*/*v*) and the absorbance was measured at 540 nm using a microplate reader (Tecan, Switzerland). The percentage of cell viability was calculated as ((a − b)/(c − b)) × 100, where a, b, and c were the absorbance of cells treated with an extract, absorbance of blank medium, and absorbance of cell control, respectively. The half-maximal cytotoxic concentration (CC_50_) was determined from the plot of percentages of cell viability versus concentrations of extract. The assay was performed in triplicate.

### 3.4. Cytopathic Effect Inhibitory Assay

The cytopathic effect inhibitory effect of each extract was assessed using the method of Chan et al. [67] with modifications. Vero cells (40,000 cells/well) were grown at 37 °C and 5% CO_2_ for 24 h. Based on the results from the cytotoxic assay, only non-toxic concentrations of the extracts were used, and two-fold serially diluted in the maintenance medium to produce six concentrations for evaluation. Two modes were used to introduce the extracts into 96-well microplates; in the concurrent mode, 100 µL each of the extract solution and the virus inoculum at a density of multiplicity of infection (MOI) of one were added to the cells simultaneously while in the non-concurrent mode, the cells were treated with the extracts for 24 h before the addition of the virus inoculum (MOI = 1). The final concentration range of each extract varied, ranging from 0.08–2.50 µg/mL to 20–640 µg/mL, depending on the extract. The same final concentration range was used for each extract in both modes. The treated Vero cells in both modes were further incubated at 37 °C and 5% CO_2_ for 72 h, and the cell viability was measured as described previously. Chloroquine diphosphate (MP Biomedicals, Santa Ana, CA, USA) with a concentration range of 0.20–6.40 µg/mL was used as a positive control [53]. Other controls in the assay included virus control (untreated, infected) and cell control (untreated, uninfected). Percentage of cell viability was calculated as ((x − y)/(z − y)) × 100, where x, y, and z were the absorbance of cells treated with extract and virus, absorbance of virus control, and absorbance of cell control, respectively. The half-maximal effective concentration (EC_50_) of an extract was determined from a curve colligating the percentages of cell viability to the concentrations of the extract. Results were obtained from triplicate assays.

### 3.5. Quantification of Chikungunya Virus RNA Copy Number

Extracts with concentrations showing cell viability ≥90% in the cytopathic effect inhibitory assay were selected for analysis using real-time RT-PCR [70]. The viral loads for the positive control, virus control, and viral inoculum (MOI = 1) were quantified as well. The quantification was performed in triplicate.

#### 3.5.1. Viral RNA Extraction

The cytopathic effect inhibitory assay was repeated for the extract concentrations showing ≥90% cell viability. The supernatant pooled from the medium and lyzed cells (treated and infected) was harvested and subjected to viral RNA extraction. The extraction was performed using the Invisorb^®^ Spin Virus RNA Mini Kit (Invitrogen, Waltham, MA, USA) according to the manufacturer’s instructions. The eluted RNA was stored at −80 °C pending real-time RT-PCR.

#### 3.5.2. Generation of Viral RNA Standard

The chikungunya virus RNA standard was generated through in vitro synthesis of RNA transcripts from DNA templates using the MAXIscript*^®^* in vitro transcription kit (Invitrogen, Waltham, MA, USA). A forward primer (CHIK/E1/10367/+) with an incorporated T7 promoter sequence (5-TAATACGACTCACTATAGGGCTCATACCGCATCCGCATCAG-3′) was used. The sequence of the reverse primer was 5′-ACATTGGCCCCACAATGAATTTG-3′ (CHIK/E1/10495/-). One µg of PCR product (DNA template) was subjected to in vitro transcription at 37 °C for 1 h. The volumes of transcription buffer, ribonucleotide solutions, and RNA polymerase were applied according to the instructions of kit’s manufacturer. In order to remove the template DNA, the transcribed products were treated with 1 µL of DNase I and incubated at 37 °C for 30 min. The DNase activity was terminated by adding 1 µL of 0.5 mol/L ethylenediaminetetraacetic acid and heat-deactivated at 95 °C for 10 min. The unincorporated ribonucleotides were removed by ammonium acetate/ethanol precipitation. The resulted RNA pellet was dissolved in diethyl pyrocarbonate-treated water (Bio-Basic, Markham, ON, Canada) and stored at −80 °C. The concentration of the synthesized viral RNA was determined using a nanospectrophotometer (Implen, Westlake Village, CA, USA) and converted to molecular copies [71].

#### 3.5.3. One-Step SYBR Green-Based Real-Time RT-PCR

The real-time RT-PCR was conducted using the iScript™ One-Step RT-PCR kit (BioRad, Hercules, CA, USA) with the Rotor-Gene Q Real Time PCR machine (Qiagen, Germantown, MD, USA). The samples were assayed in a 25 µL reaction containing 2 µmol/L of forward primer (CHIK/E1/10367/+: 5′-CTCATACCGCATCCGCATCAG-3′), 2 µmol/L of reverse primer (CHIK/E1/10495/-: 5′-ACATTGGCCCCACAATGAATTTG-3′), 5 µL of extracted RNA, 0.25 µL of RNA transcriptase, and 12.5 µL of SYBR^®^ Green Premix. The concentrations of Taq polymerase, buffer, dNTPs, and Mg^2+^ used were based on the recommendations of the manufacturer. The RT-PCR thermal cycling condition comprised 30 min of reverse transcription step at 50 °C, 15 min of initial denaturation at 95 °C, followed by 40 cycles of amplification steps of denaturation at 95 °C for 30 s, annealing at 55.8 °C for 45 s, extension at 72 °C for 60 s, and a final extension at 72 °C for 10 min. A melting curve was generated after the amplification step at 70–99 °C. A standard curve was constructed by using the synthesized RNA standard with copy numbers ranging from 10^0^ to 10^10^.

### 3.6. Data Analysis

The selectivity index (SI) of an extract was calculated as the ratio of CC_50_ to EC_50_ of the extract. The data of cytopathic effect inhibitory assay were analyzed for statistical significance using one-way analysis of variance (ANOVA) with a significance level (α) of 0.05. Tukey’s test or Dunnett’s test was used in the post-hoc analysis. The data obtained from viral load quantification were analyzed using independent-Student’s *t*-test. The normality and homogeneity of variance of data were assessed using the Shapiro-Wilk test and Levene’s test, respectively. All statistical analyses were performed using the IBM SPSS Statistics for Windows Version 23.0 software (IBM Corp., Armonk, NY, USA).

## 4. Conclusions

For this research 132 extracts from 21 medicinal plant species were evaluated for cytopathic effect inhibitory activity against the chikungunya virus using concurrent and non-concurrent sample introduction modes. The inhibitory effect of the extracts was dependent on plant species, extract concentration, type of extractant, and sample introduction mode. More extracts were found to have strong inhibitory activity in the concurrent mode than in the non-concurrent mode. Analysis of selected 21 extracts from eight plants with a strong inhibitory activity using real-time RT-PCR indicates that the active extracts targeted the chikungunya virus life cycle at different stages, including inhibition of virus entry into Vero cells, blocking the release of viral progeny from the cells, and virucidal effect on the virus. Some of the medicinal plants such as *G. bicolor*, *H. sibthorpioides* and *O. americanum* even possessed multiple antiviral mechanisms. The bioactive compounds in the plant extracts could be isolated and characterized as lead compounds for potential pharmaceutical developments into anti-chikungunya virus drugs. The plant extracts could also be evaluated against other viruses such as dengue virus, which causes another endemic mosquito-borne disease in Malaysia. The results of this study reiterated the fact that medicinal plant extracts contain many phytochemicals with biological activities. Medicinal plants could be explored as an accessible and sustainable source of chemotherapeutic agents for the treatment of emerging or re-emerging viral diseases. More collaborative efforts are needed to pursue the exploration of medicinal plants for human health benefits.

## Figures and Tables

**Table 1:** Details and uses of selected medicinal plants.

Plant Name	Family	Vernacular Name	Part Used	Medicinal or Folkloric Uses	Voucher Number
*Ailanthus triphysa* (Dennst.) Alston	*Simaroubaceae*	White siris	Leaf	Hypertension, bronchitis, dysentery [32]	UTAR/FSC/11/004
*Archidendron jiringa* (Jack) I.C.Nielsen	*Leguminosae*	Djengkol bean	Seed	Bladder stones, hypertension, diabetes [33]	Nil
*Arundina graminifolia* (D.Don) Hochr.	*Orchidaceae*	Grass orchid	Leaf	Snake bites, rheumatism, stomachache [34]	UTAR/FSC/10/011
*Azadirachta indica* A.Juss.	*Meliaceae*	Neem	Leaf	Leprosy, skin ulcers, biliousness, epistaxis, eye problem, anorexia, intestinal worms [35]	UTAR/FSC/11/001
*Basella alba* L.	*Basellaceae*	Ceylon spinach, Malabar spinach	Leaf	Constipation, liver and urinary diseases, catarrh, gonorrhea, boils, sore throat, hypertension, burns [36]	UTAR/FSC/10/014
*Beta vulgaris* L.	*Amaranthaceae*	Beetroot	Root	Dandruff, decreased libido, constipation, joint pain [37]	Nil
*Clinacanthus nutans* (Burm.f.) Lindau	*Acanthaceae*	Sabah snake grass	Leaf	Diabetes, dysentery, eye diseases, skin rashes, allergic responses, insect and snake bites [38]	UTAR/FSC/11/003
*Curcuma longa* L.	*Zingiberaceae*	Turmeric	Rhizome	Stomachic and intestinal diseases, arthritis, gall stones, emmenagogue, bruise, as a tonic [39]	Nil
*Diodella sarmentosa* (Sw.) Bacigalupo & Cabral ex Borhidi	*Rubiaceae*	Tropical buttonweed	Leaf and stem	Ulcers, snake bite, rheumatic inflammatory disorders, venereal diseases [40]	UTAR/FSC/10/018
*Diplazium esculentum* (Retz.) Sw.	*Athyriaceae*	Vegetable fern	Leaf and stem	Constipation, hypertension [41]	UTAR/FSC/10/023
*Ficus deltoidea* Jack	*Moraceae*	Mistletoe fig	Leaf	Wounds, rheumatism, sores, as an after-birth tonic [42]	UTAR/FSC/10/021
*Gynura bicolor* (Roxb. ex Willd.) DC.	*Compositae*	Okinawa spinach	Leaf	Blood circulation improvement, diabetes, dysmenorrhea, hemoptysis, post-labor recovery [43]	UTAR/FSC/11/005
*Homalocladium platycladum* (F.Muell.) L.H.Bailey.	*Polygonaceae*	Centipede plant	Stem	Skin swelling, sores, insect and snake bites, fracture injuries, fever [44]	UTAR/FSC/10/017
*Hydrocotyle sibthorpioides* Lam.	*Araliaceae*	Lawn marsh pennywort	Whole plant	Cough, cold, fever, zoster, eczema, hepatitis, jaundice [45]	UTAR/FSC/10/019
*Manilkara zapota* (L.) P.Royen	*Sapotaceae*	Sapodilla, Ciku	Fruit	Diarrhea, pulmonary complaints [46]	Nil
*Ocimum americanum* L.	*Lamiaceae*	Hoary basil	Leaf	Fever, colds, dysentery, toothache, migraine [47]	UTAR/FSC/10/013
*Parkia speciosa* Hassk.	*Leguminosae*	Stink bean	Seed and pod	Urinary infections, diabetes, loss of appetite [48]	UTAR/FSC/10/015
*Petroselinum crispum* (Mill.) Fuss	*Apiaceae*	Parsley	Leaf and stem	Skin diseases, eczema, hypertension, diabetes, nosebleed, constipation pain, baldness [49]	UTAR/FSC/10/024
*Salacca zalacca* (Gaertn.) Voss	*Arecaceae*	Salak	Fruit	Diabetes [50]	Nil
*Sechium edule* (Jacq.) Sw.	*Cucurbitaceae*	Chayote	Leaf and stem	Kidney stones, hypertension [51]	UTAR/FSC/10/022
*Strobilanthes crispus* (L.) Blume	*Acanthaceae*	Yellow strobilanthus, “kejibeling”	Leaf	Kidney stones, enhance immune system [52]	UTAR/FSC/10/020

**Table 2:** Classification of cytopathic effect inhibitory activity of each medicinal plant extract for the concurrent and non-concurrent modes.

Plant	Part	Concurrent Mode						Non-Concurrent Mode					
	Extract ^#^	HX	CF	EA	EN	MN	WT	HX	CF	EA	EN	MN	WT
*Ailanthus triphysa*	Leaf	W	W	W	W	W	W	W	W	W	W	W	W
*Archidendron jiringa*	Seed	W	I	W	W	W	W	W	I	W	W	W	W
*Arundina graminifolia*	Leaf	W	W	W	W	W	W	W	I	I	W	W	W
*Azadirachta indica*	Leaf	I	S	S	S	S	W	W	W	W	W	W	W
*Basella alba*	Leaf	W	I	W	W	W	W	I	I	I	W	W	W
*Beta vulgaris*	Root	W	W	W	W	W	W	W	W	W	W	W	W
*Clinacanthus nutans*	Leaf	W	S	S	S	S	W	W	W	W	W	I	W
*Curcuma longa*	Rhizome	W	I	W	W	W	I	W	W	W	W	W	W
*Diodella sarmentosa*	Leaf and stem	W	S	S	S	W	W	W	I	W	I	W	W
*Diplazium esculentum*	Leaf and stem	W	S	S	S	S	W	W	W	W	I	W	W
*Ficus deltoidea*	Leaf	W	S	I	S	W	W	W	I	S	S	S	W
*Gynura bicolor*	Leaf	W	S	S	S	S	S	W	W	I	W	W	W
*Homalocladium platycladum*	Stem	W	S	S	S	I	S	W	W	I	W	W	W
*Hydrocotyle sibthorpioides*	Whole plant	W	S	I	W	S	W	W	W	W	W	W	W
*Manilkara zapota*	Fruit	W	W	W	W	W	W	W	W	W	W	W	W
*Ocimum americanum*	Leaf	I	S	S	S	S	S	W	W	W	W	W	W
*Parkia speciosa*	Pod	W	I	I	W	W	W	W	W	W	W	W	W
*Parkia speciosa*	Seed	W	W	W	W	W	W	W	W	W	W	W	W
*Petroselinum crispum*	Leaf and stem	W	S	S	S	W	W	W	W	W	I	W	W
*Salacca zalacca*	Fruit	W	W	W	W	W	W	W	W	W	W	W	W
*Sechium edule*	Leaf and stem	W	S	S	S	W	W	W	W	I	I	W	W
*Strobilanthes crispus*	Leaf	W	S	I	S	S	W	W	W	W	W	W	W

^#^ HX: hexane; CF: chloroform; EA: ethyl acetate; EN: ethanol; MN: methanol; WT: water. S: strong inhibitory activity when cell viability ≥70%; I: intermediate inhibitory activity when cell viability is 31–69%; W: weak inhibitory activity when cell viability ≤30%.

**Table 3:** Selectivity indices and viral RNA copy numbers for the selected active medicinal plant extracts against chikungunya virus.

Plant	Extract	Half-Maximal Cytotoxic Concentration, CC_50_ (µg/mL)	Mode ^	Half-Maximal Effective Concentration, EC_50_ (µg/mL)	Selectivity Index (= CC_50_/EC_50_)	Viral RNA Copy Number (Molecules/µL)	Log Reduction ^#^
*Clinacanthus nutans*	Chloroform	602.67 ± 9.29	C	120.67 ± 4.62	4.99	7.75 × 10^9^ ± 1.69 × 10^9^ *	0.89
	Ethyl acetate	133.00 ± 9.17	C	9.93 ± 0.91	13.39	1.68 × 10^10^ ± 0.51 × 10^10^ *	0.55
	Ethanol	>640	C	31.30 ± 0.95	>20.45	8.72 × 10^9^ ± 1.25 × 10^9^ *	0.83
*Diodella sarmentosa*	Ethyl acetate	203.33 ± 6.11	C	8.33 ± 0.57	24.40	1.83 × 10^5^ ± 1.07 × 10^5^ * ^†^	5.51
*Diplazium esculentum*	Chloroform	99.00 ± 3.61	C	6.80 ± 0.26	14.56	4.23 × 10^4^ ± 0.59 × 10^4^ * ^†^	6.15
	Ethyl acetate	184.33 ± 9.24	C	14.07 ± 0.06	13.10	1.38 × 10^4^ ± 0.62 × 10^4^ * ^†^	6.63
	Ethanol	220.67 ± 1.53	C	14.30 ± 0.20	15.43	3.40 × 10^4^ ± 1.02 × 10^4^ * ^†^	6.24
	Methanol	461.00 ± 1.73	C	29.70 ± 0.60	15.52	1.12 × 10^9^ ± 0.11 × 10^9^ * ^†^	1.73
*Ficus deltoidea*	Ethanol	>640	NC	15.20 ± 0.20	>42.10	4.42 × 10^6^ ± 2.71 × 10^6 †^	4.13
*Gynura bicolor*	Chloroform	117.67 ± 9.50	C	3.65 ± 0.06	32.21	3.50 × 10^9^ ± 1.18 × 10^9^ * ^†^	1.23
	Ethyl acetate	31.33 ± 4.16	C	1.91 ± 0.03	16.43	3.71 × 10^8^ ± 2.90 × 10^8 †^	2.21
	Ethanol	55.00 ± 3.46	C	3.62 ± 0.10	15.18	2.33 × 10^5^ ± 0.58 × 10^5^ * ^†^	5.41
	Water	> 640	C	244.67 ± 4.73	>2.62	3.29 × 10^5^ ± 1.78 × 10^5 †^	5.26
*Hydrocotyle sibthorpioides*	Ethanol	610.33 ± 9.50	C	95.33 ± 2.47	6.40	4.01 × 10^10^ ± 1.54 × 10^10^ *	0.17
	Water	-	C	394.00 ± 6.93	-	4.39 × 10^5^ ± 2.74 × 10^5 †^	5.13
*Ocimum americanum*	Chloroform	86.33 ± 4.73	C	3.61 ± 0.11	23.92	5.50 × 10^5^ ± 0.75 × 10^5 †^	5.03
	Ethyl acetate	60.83 ± 2.02	C	1.37 ± 0.06	4.45	3.57 × 10^5^ ± 0.26 × 10^5 †^	5.22
	Ethanol	226.33 ± 9.87	C	1.33 ± 0.10	170.18	1.71 × 10^10^ ± 0.48 × 10^10^ *	0.54
	Methanol	>640	C	21.93 ± 0.84	>29.18	7.81 × 10^9^ ± 2.32 × 10^9^ *	0.88
*Sechium edule*	Ethyl acetate	100.67 ± 9.29	C	2.71 ± 0.25	37.10	7.43 × 10^9^ ± 2.79 × 10^9^ *	0.90
	Ethanol	-	C	90.33 ± 0.28	-	1.14 × 10^10^ ± 0.16 × 10^10^ *	0.72
Chloroquine	-	16.33 ± 0.76	NC	9.05 ± 0.05	2.89	1.68 × 10^6^ ± 0.49 × 10^6^ * ^†^	4.55
			C	1.92 ± 0.13 **	13.65	3.94 × 10^5^ ± 0.70 × 10^5 †^	5.18
Virus inoculum	-	-	-	-	-	6.25 × 10^5^ ± 2.09 × 10^5^	-
Virus control	-	-	-	-	-	5.96 × 10^10^ ± 3.33 × 10^10^	-

Values are expressed as mean ± standard deviation (n = 3). ^^^ C: concurrent mode; NC: non-concurrent mode. ^#^ compared with virus control. * significantly different (*p* < 0.05) from the virus inoculum by independent-samples *t*-test. ^†^ significantly different (*p* < 0.05) from the virus control by independent-samples *t*-test. ** significantly different (*p* < 0.001) from the non-concurrent mode by independent-samples *t*-test.

## Data Availability

All the data generated from this study have been provided in the main text.

## References

[1] Smith D.W., Mackenzie J.S., Frolov I.V., Weaver S.C., Richman D.D., Whitley R.J., Hayden F.G. (2017). Alphaviruses. Clinical Virology.

[2] Ross R.W. (1956). The Newala epidemic: III. The virus: Isolation, pathogenic properties and relationship to the epidemic. J. Hyg..

[3] Vairo F., Haider N., Kock R., Ntoumi F., Ippolito G., Zumla A. (2019). Chikungunya: Epidemiology, pathogenesis, clinical features, management, and prevention. Infect. Dis. Clin. N. Am..

[4] Silva L.A., Dermody T.S. (2017). Chikungunya virus: Epidemiology, replication, disease mechanisms, and prospective intervention strategies. J. Clin. Investig..

[5] Centers for Disease Control and Prevention Chikungunya Virus. https://www.cdc.gov/chikungunya/index.html.

[6] Puntasecca C.J., King C.H., LaBeaud A.D. (2021). Measuring the global burden of chikungunya and Zika viruses: A systematic review. PLoS Negl. Trop. Dis..

[7] European Centre for Disease Prevention and Control Communicable Disease Threats Report, Week 51, 13–19 December 2020. https://www.ecdc.europa.eu/en/publications-data/communicable-disease-threats-report-13-19-december-2020-week-51.

[8] Ministry of Health Malaysia Kenyataan Akhbar Ketua Pengarah Kesihatan Malaysia Mengenai Situasi Semasa Deman Denggi, Zika Dan Chikungunya Di Malaysia—Minggu Ke 51/2020. https://www.moh.gov.my/index.php/database_stores/store_view_page/17/1734.

[9] Ferreira F.C., da Silva A.S., Recht J., Guaraldo L., Moreira M.E., de Siqueira A.M., Gerardin P., Brasil P. (2021). Vertical transmission of chikungunya virus: A systematic review. PLoS ONE.

[10] Natrajan M.S., Rojas A., Waggoner J.J. (2019). Beyond fever and pain: Diagnostic methods for chikungunya virus. J. Clin. Microbiol..

[11] Tanabe I.S.B., Tanabe E.L.L., Santos E.C., Martins W.V., Araújo I.M.T.C., Cavalcante M.C.A., Lima A.R.V., Câmara N.O.S., Anderson L., Yunusov D. (2018). Cellular and molecular immune response to chikungunya virus infection. Front. Cell. Infect. Microbiol..

[12] Van Aalst M., Nelen C.M., Goorhuis A., Stijnis C., Grobusch M.P. (2017). Long-term sequelae of chikungunya virus disease: A systematic review. Travel Med. Infect. Dis..

[13] Powers A.M. (2017). Vaccine and therapeutic options to control chikungunya virus. Clin. Microbiol. Rev..

[14] Gao S., Song S., Zhang L. (2019). Recent progress in vaccine development against chikungunya virus. Front. Microbiol..

[15] Hucke F.I.L., Bugert J.J. (2020). Current and promising antivirals against chikungunya virus. Front. Public Health.

[16] World Health Organization (1993). Guidelines on the Conservation of Medicinal Plants.

[17] Zaynab M., Fatima M., Abbas S., Sharif Y., Umair M., Zafar M.H., Bahadar K. (2018). Role of secondary metabolites in plant defense against pathogens. Microb. Pathog..

[18] Prinsloo G., Marokane C.K., Street R.A. (2018). Anti-HIV activity of southern African plants: Current developments, phytochemistry and future research. J. Ethnopharmacol..

[19] Salehi B., Kumar N.V.A., Şener B., Sharifi-Rad M., Kılıç M., Mahady G.B., Vlaisavljevic S., Iriti M., Kobarfard F., Setzer W.N. (2018). Medicinal plants used in the treatment of human immunodeficiency virus. Int. J. Mol. Sci..

[20] Akram M., Tahir I.M., Shah S.M.A., Mahmood Z., Altaf A., Ahmad K., Munir N., Daniyal M., Nasir S., Mehboob H. (2018). Antiviral potential of medicinal plants against HIV, HSV, influenza, hepatitis, and coxsackievirus: A systematic review. Phytother. Res..

[21] Mohan S., Elhassan Taha M.M., Makeen H.A., Alhazmi H.A., Al Bratty M., Sultana S., Ahsan W., Najmi A., Khalid A. (2020). Bioactive natural antivirals: An updated review of the available plants and isolated molecules. Molecules.

[22] Geng C.A., Chen J.J. (2018). The progress of anti-HBV constituents from medicinal plants in China. Nat. Prod. Bioprospect..

[23] Saleh M.S.M., Kamisah Y. (2020). Potential medicinal plants for the treatment of dengue fever and severe acute respiratory syndrome-coronavirus. Biomolecules.

[24] Frederico É.H.F.F., Cardoso A.L.B.D., Moreira-Marconi E., de Sá-Caputo D.D.C., Guimarães C.A.S., Dionello C.D.F., Morel D.S., Paineiras-Domingos L.L., de Souza P.L., Brandão-Sobrinho-Neto S. (2017). Anti-viral effects of medicinal plants in the management of dengue: A systematic review. Afr. J. Tradit. Complement. Altern. Med..

[25] Chan Y.S., Khoo K.S., Sit N.W. (2016). Investigation of twenty selected medicinal plants from Malaysia for anti-Chikungunya virus activity. Int. Microbiol..

[26] Ledoux A., Cao M., Jansen O., Mamede L., Campos P.E., Payet B., Clerc P., Grondin I., Girard-Valenciennes E., Hermann T. (2018). Antiplasmodial, anti-chikungunya virus and antioxidant activities of 64 endemic plants from the Mascarene Islands. Int. J. Antimicrob. Agents.

[27] Weber C., Sliva K., von Rhein C., Kümmerer B.M., Schnierle B.S. (2015). The green tea catechin, epigallocatechin gallate inhibits chikungunya virus infection. Antivir. Res..

[28] Mounce B.C., Cesaro T., Carrau L., Vallet T., Vignuzzi M. (2017). Curcumin inhibits Zika and chikungunya virus infection by inhibiting cell binding. Antivir. Res..

[29] Kaur P., Thiruchelvan M., Lee R.C., Chen H., Chen K.C., Ng M.L., Chu J.J. (2013). Inhibition of chikungunya virus replication by harringtonine, a novel antiviral that suppresses viral protein expression. Antimicrob. Agents Chemother..

[30] Maria John K.M., Harnly J., Luthria D. (2018). Influence of direct and sequential extraction methodology on metabolic profiling. J. Chromatogr. B.

[31] Cowan M.M. (1999). Plant products as antimicrobial agents. Clin. Microbiol. Rev..

[32] Thongnest S., Boonsombat J., Prawat H., Mahidol C., Ruchirawat S. (2017). Ailanthusins A-G and nor-lupane triterpenoids from *Ailanthus triphysa*. Phytochemistry.

[33] Shukri R., Mohamed S., Mustapha N.M., Hamid A.A. (2011). Evaluating the toxic and beneficial effects of jering beans (*Archidendron jiringa*) in normal and diabetic rats. J. Sci. Food Agric..

[34] Zhang X., Chen W., Du Y., Su P., Qiu Y., Ning J., Liu M. (2021). Phytochemistry and pharmacological activities of *Arundina graminifolia* (D.Don) Hochr. and other common *Orchidaceae* medicinal plants. J. Ethnopharmacol..

[35] Saleem S., Muhammad G., Hussain M.A., Bukhari S.N.A. (2018). A comprehensive review of phytochemical profile, bioactives for pharmaceuticals, and pharmacological attributes of *Azadirachta indica*. Phytother. Res..

[36] Deshmukh S.A., Gaikwad D.K. (2014). A review of the taxonomy, ethnobotany, phytochemistry and pharmacology of *Basella alba* (*Basellaceae*). J. Appl. Pharm. Sci..

[37] Hamedi S., Honarvar M. (2019). *Beta vulgaris—*A mini review of traditional uses in Iran, phytochemistry and pharmacology. Curr. Drug Discov. Technol..

[38] Kamarudin M.N.A., Sarker M.M.R., Kadir H.A., Ming L.C. (2017). Ethnopharmacological uses, phytochemistry, biological activities, and therapeutic applications of *Clinacanthus nutans* (Burm. f.) Lindau: A comprehensive review. J. Ethnopharmacol..

[39] Ayati Z., Ramezani M., Amiri M.S., Moghadam A.T., Rahimi H., Abdollahzade A., Sahebkar A., Emami S.A. (2019). Ethnobotany, phytochemistry and traditional uses of *Curcuma* spp. and pharmacological profile of two important species (*C. longa* and *C. zedoaria*): A review. Curr. Pharm. Des..

[40] Akah P.A., Orisakwe O.E., Gamaniel K.S., Shittu A. (1998). Evaluation of Nigerian traditional medicines: II. Effects of some Nigerian folk remedies on peptic ulcer. J. Ethnopharmacol..

[41] Abe R., Ohtani K. (2013). An ethnobotanical study of medicinal plants and traditional therapies on Batan Island, the Philippines. J. Ethnopharmacol..

[42] Bunawan H., Amin N.M., Bunawan S.N., Baharum S.N., Mohd Noor N. (2014). *Ficus deltoidea* Jack: A review on its phytochemical and pharmacological importance. Evid. Based Complement. Alternat. Med..

[43] Teoh W.Y., Sim K.S., Moses Richardson J.S., Abdul Wahab N., Hoe S.Z. (2013). Antioxidant capacity, cytotoxicity, and acute oral toxicity of *Gynura bicolor*. Evid. Based Complement. Altern. Med..

[44] Siriwatanametanon N., Fiebich B.L., Efferth T., Prieto J.M., Heinrich Michael M. (2010). Traditionally used Thai medicinal plants: In vitro anti-inflammatory, anticancer and antioxidant activities. J. Ethnopharmacol..

[45] Hazarika I., Mukundan G.K., Sundari P.S., Laloo D. (2021). Journey of *Hydrocotyle sibthorpioides* Lam.: From traditional utilization to modern therapeutics*—*A review. Phytother. Res..

[46] Lim T.K. (2013). Edible Medicinal and Non-Medicinal Plants: Fruits.

[47] Zengin G., Ferrante C., Gnapi D.E., Sinan K.I., Orlando G., Recinella L., Diuzheva A., Jekő J., Cziáky Z., Chiavaroli A. (2019). Comprehensive approaches on the chemical constituents and pharmacological properties of flowers and leaves of American basil (*Ocimum americanum* L.). Food Res. Int..

[48] Saleh M.S.M., Jalil J., Zainalabidin S., Asmadi A.Y., Mustafa N.H., Kamisah Y. (2021). Genus *Parkia*: Phytochemical, medicinal uses, and pharmacological properties. Int. J. Mol. Sci..

[49] Farzaei M.H., Abbasabadi Z., Ardekani M.R.S., Rahimi R., Farzaei F. (2013). Parsley: A review of ethnopharmacology, phytochemistry and biological activities. J. Tradit. Chin. Med..

[50] Saleh M.S.M., Siddiqui M.J., Mediani A., Ismail N.H., Ahmed Q.U., So’ad S.Z.M., Saidi-Besbes S. (2018). *Salacca zalacca*: A short review of the palm botany, pharmacological uses and phytochemistry. Asian Pac. J. Trop. Med..

[51] Vieira E.F., Pinho O., Ferreira I.M.P.L.V.O., Delerue-Matos C. (2019). Chayote (*Sechium edule*): A review of nutritional composition, bioactivities and potential applications. Food Chem..

[52] Zakaria S.M., Amri C.N.A.C., Shahari R. (2020). Ethnobotany and traditional knowledge of Acanthaceae in Peninsular Malaysia: A review. Pharmacogn. J..

[53] Bernard E., Solignat M., Gay B., Chazal N., Higgs S., Devaux C., Briant L. (2010). Endocytosis of chikungunya virus into mammalian cells: Role of clathrin and early endosomal compartments. PLoS ONE.

[54] Khan M., Santhosh S.R., Tiwari M., Lakshmana Rao P.V., Parida M. (2010). Assessment of in vitro prophylactic and therapeutic efficacy of chloroquine against Chikungunya virus in Vero cells. J. Med. Virol..

[55] Bourjot M., Delang L., Nguyen V.H., Neyts J., Guéritte F., Leyssen P., Litaudon M. (2012). Prostratin and 12-O-tetradecanoylphorbol 13-acetate are potent and selective inhibitors of Chikungunya virus replication. J. Nat. Prod..

[56] Scholte F.E., Tas A., Martina B.E., Cordioli P., Narayanan K., Makino S., Snijder E.J., van Hemert M.J. (2013). Characterization of synthetic Chikungunya viruses based on the consensus sequence of recent E1-226V isolates. PLoS ONE.

[57] Raghavendhar S., Tripati P.K., Ray P., Patel A.K. (2019). Evaluation of medicinal herbs for Anti-CHIKV activity. Virology.

[58] Sakdarat S., Shuyprom A., Pientong C., Ekalaksananan T., Thongchai S. (2009). Bioactive constituents from the leaves of *Clinacanthus nutans* Lindau. Bioorg. Med. Chem..

[59] Kunsorn P., Ruangrungsi N., Lipipun V., Khanboon A., Rungsihirunrat K. (2013). The identities and anti-herpes simplex virus activity of *Clinacanthus nutans* and *Clinacanthus siamensis*. Asian Pac. J. Trop. Biomed..

[60] Sookmai W., Ekalaksananan T., Pientong C., Sakdarat S., Kongyingyoes B. (2011). The anti-papillomavirus infectivity of *Clinacanthus nutans* compounds. Srinagarind Med. J..

[61] Tu S.-F., Liu R.H., Cheng Y.-B., Hsu Y.-M., Du Y.-C., El-Shazly M., Wu Y.-C., Chang F.-R. (2014). Chemical constituents and bioactivities of *Clinacanthus nutans* aerial parts. Molecules.

[62] Husin F., Chan Y.Y., Gan S.H., Sulaiman S.A., Shueb R.H. (2015). The effect of *Hydrocotyle sibthorpioides* Lam. extracts on in vitro dengue replication. Evid. Based Complement. Altern. Med..

[63] Huang Q., Zhang S., Huang R., Wei L., Chen Y., Lv S., Liang C., Tan S., Liang S., Zhuo L. (2013). Isolation and identification of an anti-hepatitis B virus compound from *Hydrocotyle sibthorpioides* Lam. J. Ethnopharmacol..

[64] Yucharoen R., Anuchapreeda S., Tragoolpua Y. (2011). Anti-herpes simplex virus activity of extracts from the culinary herbs *Ocimum sanctum* L., *Ocimum basilicum* L. and *Ocimum americanum* L. Afr. J. Biotechnol..

[65] Hanisa H., Mohd Azmi M.L., Suhaila M., Somchit M.N. (2014). In vitro anti-viral activity of *Centella asiatica* L., *Curcuma longa* L. and *Strobilanthes crispus* L. against herpes virus. Int. J. Pharma Bio Sci..

[66] Mouhajir F., Hudson J.B., Rejdali M., Towers G.H.N. (2001). Multiple antiviral activities of endemic medicinal plants used by Berber peoples of Morocco. Pharm. Biol..

[67] Chan Y.S., Ong C.W., Chuah B.L., Khoo K.S., Chye F.Y., Sit N.W. (2018). Antimicrobial, antiviral and cytotoxic activities of selected marine organisms collected from the coastal areas of Malaysia. J. Mar. Sci. Technol..

[68] Reed L.J., Muench H. (1938). A simple method of estimating fifty per cent endpoints. Am. J. Epidemiol..

[69] Chan S.M., Khoo K.S., Sit N.W. (2015). Interactions between plant extracts and cell viability indicators during cytotoxicity testing: Implications for ethnopharmacological studies. Trop. J. Pharm. Res..

[70] Ali U.H., Vasan S.S., Thayan R., Angamuthu C., Lim L.H., Sekaran S.D. (2010). Development and evaluation of a one-step SYBR-Green I-based realtime RT-PCR assay for the detection and quantification of Chikungunya virus in human, monkey and mosquito samples. Trop. Biomed..

[71] Krieg P.A. (1990). Improved synthesis of full-length RNA probe at reduced incubation temperatures. Nucleic Acids Res..

